# Towards a unified measure of general interpersonal trust

**DOI:** 10.1016/j.heliyon.2024.e40624

**Published:** 2024-11-22

**Authors:** Uyen Hoang, Braden Tanner, Dana Mahmoud-Elhaj, Jenna Holt, Muhammad Asif, Adam Feltz

**Affiliations:** Department of Psychology, University of Oklahoma, Norman, OK, USA

**Keywords:** General trust, Reliability, Confidence, Honesty, Trustworthiness

## Abstract

Trust is a well-studied attitude with many different conceptualizations, scales, and measurement techniques. However, the diversity of measurement and conceptualizations of trust can make comparisons between studies and instrument selection difficult. To help address these difficulties, we conducted 4 studies where we directly compared commonly used domain general trust measures. Study 1 (*N* = 155) compared 6 commonly used measures of trust (with a total of 10 factors reported in those measures) and submitted items in those scales to an exploratory factor analysis. We were able to identify 6 distinct, interpretable factors in those trust measures. Studies 2–4 (*Ns =* 234, 166, 355) reduced the 6 factors to 4 that were consistent with the theoretical literature: Confidence in others, belief in others’ reliability, belief in others’ honesty, and beliefs about others’ trustworthiness. Study 4 also suggested that the 4 trust factors were largely unrelated to sex, political orientation, and disgust sensitivity (discriminant validity). The 4 trust factors were associated with other, related factors such as the personality trait agreeableness (convergent validity). Some of the trust factors predicted decisions about acceptance of water recycling (predictive validity) and predicted that acceptance beyond what was predicted by disgust sensitivity, sex, and political orientation (incremental validity).

Trust is commonly held to be one critical element in many inter-personal interactions. Not surprisingly, trust has been extensively discussed both theoretically and empirically. Partially as a function of this interest, there are several empirical measures of trust. One complication with having several measures of trust is that comparisons among studies that evaluate trust can be difficult. The diversity of instruments can present problems for making an informed decision about instrument selection for studies as different scales claiming to measure the same construct do not always measure the same construct and are therefore not always interchangeable [1,2]. In other words, scales should not be arbitrarily selected and utilized in a study solely on the assumption that they all measure the same construct [3,4]. Without a direct comparison of these existing trust scales, it is challenging to understand when, where, how, and for whom to use these scales or if the scales are similar, different, or more or less efficient. We aim to begin to address these problems by comparing 6 commonly used domain general measures of trust. After applying factor analytic techniques to identify common trust factors and reducing the total number of items used to measure those factors, we identified four trust factors that are consistent with major theoretical accounts of trust: (1) confidence in others, (2) belief in other’s reliability, (3) belief in other’s honesty, and (4) beliefs about other’s trustworthiness. The final instrument includes 14 items that measured these four factors. We call the 4-factor measure the General Interpersonal Trust Scale. We provide evidence for construct validity and utility of using the instrument by showing that these factors can predict acceptance of important natural resource management techniques (e.g., acceptance of recycled water). We conclude by discussing some other possible uses of the General Interpersonal Trust Scale.

## 1 Previous theoretical and empirical work on trust

‘Trust’ is a vexed concept with no clear consensus on what it means. While there is no widespread agreement about how to define trust, there has been some theoretical consensus about some core features of trust. First, one can think of the trust relation as dyadic. In this dyadic relation, there is the trustor (the person trusting) and the trustee (the target of trusting) [5,6]. Second, trust is a cognitive state or attitude of the trustor [7]. Third, the cognitive state or attitude of the trustor has some core features. These three core features are that the trustor (1) is vulnerable to the trustee (e.g., the trustor is not confident the trustee will do what the trustee is trusted with) ([8]; Boss, 1987 [9]),: (2) has favorable attitudes about the honesty and intentions of the trustee, and (3) believes that the trustee is competent and reliable [10,11]. We call these core features confidence, honesty, and reliability respectively. It is debatable whether these features are conceptually sufficient for trust (although they may be necessary). Some theorists think that some other features are required for a person to count as trusting (e.g., the trustor believes the trustee acts with good motivations). While these theorists may be correct, additional features are contentious. Unlike the more contentious features, features 1–3 enjoy wide acceptance in the conceptual literature [11].

Conceptually, trust is often distinguished from *trustworthiness* (e.g., Ref. [7,[12], [13], [14]]). For example, Mayer et al. [7]’s proposed integrative model of organizational trust makes a clear distinction between trust and trustworthiness where perceived trustworthiness is the antecedent of trust but is not trust itself (i.e., perceived trust worthiness contributes to trust). Trustworthiness is a property of the *trustee* [7,[15], [16], [17]] and not an attitude or cognitive state of the *trustor*. It is conceptually possible for a trustor to trust a person who is not trustworthy, and it is also possible for a trustor not to trust someone who is trustworthy. There of course is a non-accidental connection between trust and trustworthiness. Those who are perceived to be trustworthy are more likely to be trusted [18,19]. That having been said, the important conceptual element is that the trustor can have attitudes and beliefs about the trustworthiness of the trustee, and this attitude is theoretically (and perhaps empirically) distinct from judgments of confidence, reliability, and honesty [20].

Given the theoretical landscape, one may expect to find diverse empirical operationalizations and measures of trust. Indeed, that is exactly what we find. One study found there were 72 identifiable, distinct definitions of trust in the empirical literature (see Castaldo, Premazzi, & Zerbini [21] for a review, also McKnight & Chervany [22]). The diversity has translated into several different instruments to measure trust. There are some common elements in the measures that allow us to categorize some of them. One major distinction in the empirical measures of trust is *generalized* and *particularized* trust. Like most concepts, the exact definitions of these kinds of trust are contested. But one way to differentiate the two is that particularized trust references some concrete or known trustee whereas generalized trust does not. To illustrate, there are several particularized trust instruments that measure “trust in *x*” where *x* is some domain or class of people (e.g., trust in science and scientists [23], trust in government [24], trust in automation [25]). Generalized trust does not measure trust in any particular, concrete entity but purports to measure tendencies to trust in general [[26], [27], [28], [29], [30]]. Some prominently used generalized trust measures are the Interpersonal Trust Scale [31], the Propensity to Trust Scale [32], the Trust Scale [33], the New Propensity to Trust Scale (Ashleigh & Dulewicz, 2012), the Trust Inventory [34], and the World Values Survey Trust Instrument (World Values Survey Association, 2014).

Unfortunately, the generalized/particularized distinction is largely where the systematic similarity ends. In this paper, we will focus on generalized trust. Generalized trust has been measured as a unidimensional trait (e.g., Rotter [31]), as 2-dimensional trait (e.g., Evans & Revelle [32]), and as a 3-dimensional trait (e.g., Ashleigh & Dulewicz (2012)). No scale, to our knowledge, measures every major conceptual element of trust discussed above. Moreover, there have been very few direct comparisons among existing trust scales to determine if the different measures estimate different factors or if those different instruments offer unique predictions. Without direct comparisons between existing scales, it is difficult to know when, where, or for whom to use the scales or if the different scales are similar, different, better, worse, or more efficient. Many have noted that this lack of direct comparisons between different trust scales has hindered cumulative progress [35].

Our aim was to make progress in understanding the relations among the diverse measures of trust. We conducted a series of studies with two major goals. The first goal was to compare existing prominent measures of generalized trust against each other to determine the degree of similarity between those measures. The second goal was to synthesize the measures to identify which factors of trust could be reliably identified. Doing so might begin to provide a common way to measure the major factors of generalized trust. To foreshadow, in four studies we identified four major components of trust identified in the theoretical literature above: confidence, reliability, honesty, and trustworthiness.

## 2 Study 1

Study 1 was designed to accomplish two goals. First, we wanted direct comparisons of commonly used generalized trust scales to estimate the degree of similarity among them. These comparisons led us to our second goal. Given the conceptual distinctions discussed above, we expected weaker correlations among some of the scales indicating the potential existence of different latent variables measured by those scales. We performed an exploratory factor analysis on all the items in the scales to help identify a core set of items to measure key conceptual components of trust.

### 2.1 Method

**Participants.** Given the anticipated length of the survey, we decided to use a University’s undergraduate subjects pool that typically has fewer dropouts and greater attention than online samples for longer studies (e.g., research suggests online surveys should be no longer than 20 min [36]). A hundred and fifty-five participants were recruited from a Midwestern University. All participants were recruited through the SONA system and the study was administered through Qualtrics survey. Twenty-five participants were excluded for not completing the survey. The mean age was 20 (*SD* = 3.47, *range* = 18–43) and 67 % (*N* = 102) were female (response options was only male or female for self-reporting of gender in all studies). The University of Oklahoma IRB approval number is 13138.

**Procedure.** We selected six instruments measuring generalized trust based on citations, and theoretical relevance to generalized trust. These 6 instruments have among highest numbers of citations according to Google Scholar at the time of writing (*N*’s = 6467; 453; 3336; 98; 216; 60,000). These 6 instruments measured a total of 10 factors. We presented each instrument as they appeared in the original sources along with the original measurement scales (i.e., Likert scales with original scale length and anchor labels). We presented all items in the 6 instruments simultaneously in random order. The instruments were:

*The Interpersonal Trust Scale* [31]. This measure is a 25-item (e.g., Most people answer public opinion polls honestly), unidimensional scale that measures generalized trust. Each item was rated on a five-point Likert scale (1 = strongly agree, 5 = strongly disagree).

*The Propensity to Trust Scale* [32]. This measure consists of two subscales. The first subscale measures generalized trust (11 items, e.g., I can get along with most people) and the second subscale measures trustworthiness (10 items, e.g., I believe that most people are basically moral) on a six-point Likert scale (1 = strongly inaccurate, 6 = strongly accurate.

*The Trust Scale* [33]. This measure is a unidimensional scale measuring generalized trust. The Trust Scale consists of six items, and participants responded on a Likert Scale (1 = strongly disagree, 5 = strongly agree).

*The New Propensity to Trust Scale* [37]. This measure consists of three subscales that measure trusting others (9 items, e.g., “Other people are out to get as much as they can for themselves”), others’ reliability (7 items, e.g., “Other people can be relied upon to do what they say they will do”), and risk aversion (4 items, e.g., “It is important to save for a rainy day”) on a seven-point Likert scale (1 = strong disagreement, 7 = strong agreement).

*The Trust Inventory* [34]. This measure consists of two factors of trust. The generalized trust subscale consists of 20 items (e.g., I have few difficulties trusting people) and the personalized trust consists of 20 items (e.g., “My partner makes me feel safe”) rated on a five-point Likert scale (1 = strongly disagree, 5 = strongly agree).

*The World Values Survey Trust Instrument* (*WVS)* (World Values Survey Association, 2014). The WVS is a unidimensional measure of generalized trust. The WVS consists of 3 items. Each item asks the respondent to select the choice that the respondent finds most correct (e.g., “Generally speaking would, you say that most people can be trusted or that you need to be very careful in dealing with people? Most people can be trusted/need to be very careful”). The trusting response was coded as ‘1’ and the non-trusting response was coded as ‘0’.

### 2.2 Results and discussion

First, we calculated correlations among the generalized trusts scales used in this study. The correlations among these scales were moderate to high in most cases (see Table 1). Generally, the scales that measured the same factors had correlations ranging from .49 to .7. For example, Couch et al.’s General Trust factor was strongly related to Evans and Revelle’s General Trust factor (*r* = .7). However, in other cases, the correlations were weak or near zero. For example, the Risk Aversion factor of Ashleigh and Dulewicz (2012) scale was not strongly correlated with any other instrument (correlation absolute value not greater than .28). But this might not be surprising because risk aversion might not be an important factor of trust. Specifically, if we think that trust is characterized by being vulnerable to others (one of the necessary conditions of trust), then trust might not correlate strongly with risk aversion (e.g., if you are a highly risk averse individual, you are not necessarily wanting to be vulnerable to others and therefore might not need to be highly trusting of others). Other facets of trust (e.g., trustworthy) were more weakly related to measures of generalized trust (e.g., Rotter’s scale and Evans and Revelle [32] scale’s Trustworthy factor *r* = .26). This pattern of results suggested that there are likely to be some similar, but also some different, latent variables measured by the different trust scales.

**Table 1:** Pearson correlations among major trust scales.

Authors	1	2	3	4	5	6	7	8	9	10
1. Rotter, Evans & Revelle	1									
2. Evans & Revelle Trust	.51∗∗	1								
3. Evans & Revelle Trustworthy	.26∗∗	.4∗∗	1							
4. Yamagishi & Yamagishi	.43∗∗	.46∗∗	.42∗∗	1						
5. Ashleigh & Dulewicz Trust	.49∗∗	.61∗∗	.18∗	.37∗∗	1					
6. Ashleigh & Dulewicz Reliable	.58∗∗	.38∗∗	.33∗∗	.34∗∗	.32∗∗	1				
7. Ashleigh & Dulewicz Risk aversion	−.15∗	−.1	.28∗∗	−.05	−.19∗	.02	1			
8. Couch et al. Personal	.27∗∗	.48∗∗	.34∗∗	.2∗	.41∗∗	.25∗∗	.10	1		
9. Couch et al. General	.5∗∗	.7∗∗	.42∗∗	.52∗∗	.59∗∗	.49∗∗	.04	.56∗∗	1	
10. World Values Survey	.24∗∗	.27∗∗	.06	.32∗∗	.17∗∗	.16∗	−.21∗	.05	.29∗∗	1
*Mean*	2.84	3.95	4.65	3.63	3.93	4.1	5.29	3.71	3.55	1.68
*SD*	0.37	0.62	0.6	0.76	0.81	0.75	0.87	0.6	0.52	0.76
Response scale	1 = Strongly disagree – 5 = Strongly agree	1 = Strongly inaccurate – 6 = Strongly accurate	1 = Strongly inaccurate – 6 = Strongly accurate	1 = Strongly disagree– 5 = Strongly agree	1 = Strong disagreement – 7 = Strong agreement	1 = Strong disagreement – 7 = Strong agreement	1 = Strong disagreement – 7 = Strong agreement	1 = Strongly disagree – 5 = Strongly agree	1 = Strongly disagree – 5 = Strongly agree	1 = Low trust – 3 = High trust

*Note. ∗p* < .05, *∗∗p* < .01.

To begin to identify potential latent variables, we conducted an exploratory factor analysis entering all the items in all the scales at once. While different items have slightly different response scales (see Table 1), exploratory factor analysis and correlational analysis are not typically sensitive to differences in response scales [38]. The exploratory factor analysis used parallel analysis with principal axis extraction and oblimin rotation. The parallel analysis’s simulated eigenvalue to retain factors was 1.65 and above. As expected with many indicator items (i.e., 115), this technique identified many factors (*N* = 10) (full factor analytic results available on the OSF for this study: https://osf.io/pwxtf). Four factors accounted for less than 4 % of the total variance and were difficult to interpret conceptually. The other 6 factors accounted for greater than 4 % of the total variance and were more easily conceptually interpretable. After visually inspecting these 6 factors and examining the content of the items in each factor, we name these 6 factors: Trust in Partners (7 % of total variance), Reliability (5 %), General Trusting (4 %), Trustworthiness (4 %), Honesty (4 %), and Confidence (4 %).

## 3 Study 2

Given the exploratory nature of Study 1, Study 2 was designed to replicate and further refine the items identified in the exploratory factor analysis from Study 1.

### 3.1 Method

**Participants.** Because the length of the survey was anticipated to be substantially shorter (i.e., 29 items) than Study 1, we decide to recruit participants from Amazon’s Mechanical Turk. Samples taken from Amazon’s Mechanical Turk have been shown to be adequate for these types of tasks and are often more representative of the general U.S. population compared to traditional sources (e.g., university subjects pool) [[39], [40], [41], [42], [43], [44]]. Two hundred and thirty-four participants were recruited from Amazon’s Mechanical Turk sample. Thirty-six participants were excluded for not completing the survey. One hundred and twenty-five were male (63 %) and the mean age was 34.19, *SD* = 10.94.

**Procedure.** Based on the results of Study 1, we reduced the total items in the scale in two ways. We first visually examined factor loadings to retain items from Study 1 that (a) had the strongest factor loadings (>.35) and (b) relatively small cross-loadings (<.35) [45]. We supplemented this analysis with Item Response Theory (IRT) analysis for graded response model which largely yielded similar results in terms of item retention. Results are uploaded under Supplemental Materials on our Open Science Framework page (https://doi.org/10.17605/OSF.IO/PWXTF). Second, we also removed all items that loaded on uninterpretable factors, on the General Trust factor, and the Trust in Partners factor. We removed the items from the General Trust factor because the other 4 factors are generally thought to be aspects of and possibly constitutive of general trust (see discussion below for the potential existence of a higher order trust factor). We were primarily interested in factors involved in trust. We removed items from the Trust in Partners factor because items in that factor reference a concrete other (e.g., partner) making it conceptually more akin to a domain specific factor and not a domain general factor of trust. Finally, we made WVS question 2 into two different questions since it appeared to be problematically double-barreled. This left 29 items that were randomly presented to participants all on one page (items are listed in Table 2).

**Table 2:** Retained factors, items, and factors loadings from the Exploratory Factor Analysis in Study 1.

Origin		Item	Factor Loading
*Factor 1. Other’s reliability*			
1	Ashleigh R2	Other people cannot be relied upon	.61
2	Ash R6	Other people who act in friendly way towards me are disloyal behind my back	.62
3	Evans R17	I feel short-changed in life	.61
4	Ash R3	I have little faith in other people’s promises.	.62
5	Ash R9	Other people let you down.	.48
6	Couch R20	I would admit to being more than a little paranoid about people I meet	.42
7	Rotter 14	Most elected official are really sincere in their campaign promises.	.36
*Factor 2. Trustworthiness*			
8	Yamagishi 3	Most people are basically good and kind.	.65
9	Yamagishi 2 Couch 20	Most people are trustworthy.	.57
10	Yamagishi 1	Most people are basically honest.	.48
11	Rotter 20	Most idealists are sincere and practice what they preach.	.44
12	Evans 13	I believe that people are basically moral.	.43
13	Yamagishi 6	Most people will respond in kind when they are trusted by others.	.38
*Factor 3. Good Intentions*			
14	Rotter R10	It is safe to believe that in spite of what people say most people are primarily interested in their own welfare.	.62
15	Ashleigh R8	Other people are only concerned with their own well-being.	.61
16	Ashleigh R4	Other people are primarily interested in their own welfare despite what they say.	.58
17	Ashleigh R1	Other people are out to get as much as they can for themselves.	.58
18	Rotter R19, Ashleigh 5	In these competitive times one has to be alert or someone is likely to take advantage of you	.45
19	Rotter 22	Most students in school would not cheat even if they were sure of getting away with it.	.43
20	WVS 2∗	Most of the time, people try to be helpful. Most of the time, people are mostly looking out for themselves.	.39
*Factor 4. Confidence*			
21	Couch 2	I tend to be accepting of others.	.59
22	Couch 30	I have a lot of faith in people I know	.48
23	Couch 35	When it comes to people I know, I am believing and accepting.	.45
24	Rotter 25 Ashleigh 2	Most people answer public opinion polls honestly.	.44
25	Couch 5	I do not worry that my partner will leave me.	.44
26	Evans 8	I believe that laws should be strictly enforced.	.43
27	Evans 4	I can get along with most people.	.43
28	Evans 9	I value cooperation over competition.	.4

*Note*. ∗ Items were originally one item but were subsequently turned into two items to avoid problematic double-barreled or complication questions.

### 3.2 Results and discussion

The same basic structure found in Study 1 was found in Study 2, although the factor structures and indicator items became clearer. We used the Kaiser criterion to retain factors (eigenvalues greater than 1) [46] and principal axis factoring. This method retained 6 factors. In this exploratory factor analysis, there was consistency among some items, but other items were problematic. Items 3 and 7 loaded on factors that were not the same as in Study 1. We also identified items with cross-loadings greater than .3: items 13, 14, 30, 33, 25, and 44. Because we were interested in identifying a short measure, we conducted a second exploratory factor analyses on the remaining items after excluding items 3, 7, 13, 14, 30, 33, 25, and 44. In the second exploratory factor analysis we identified items with relatively weak factor loadings (<.6). Those items were 17, 29, 39 and we eliminated them from subsequent analyses. After this process, 14 items remained spanning four factors we identified as: Confidence, Reliability, Honesty, and Trustworthiness (see Table 3). We also supplemented this process with IRT analysis for graded response models. Results are uploaded under Supplemental Materials on our Open Science Framework page (https://doi.org/10.17605/OSF.IO/PWXTF).

**Table 3:** Final general interpersonal trust scale items.

Origin		Item
*Factor 1: Confidence in others*		
1	(Rotter 25, Ashleigh 12)	Most people answer public opinion polls honestly.
2	(Evans, Revelle 14)	I can get along with most people.
3	(Couch 2)	I tend to be accepting of others.
*Factor 2: Belief in other’s reliability*		
4	(Evans, Revelle 17)	I feel short-changed in life.
5	(Ashleigh 2)	Other people cannot be relied upon.
6	(Ashleigh 9)	Other people let you down.
*Factor 3: Belief in other’s honesty*		
7	(Rotter 10)	It is safe to believe that in spite of what people say most people are primarily interested in their own welfare.
8	(WVS 2)	Most of the time, people are mostly looking out for themselves.
9	(Ashleigh 4)	Other people are primarily interested in their own welfare despite what they say.
10	(Ashleigh 8)	Other people are only concerned with their own well-being.
*Factor 4: Belief in other’s trustworthiness*		
11	(Evans, Revelle 13)	I believe that people are basically moral.
12	(Yamagishi 1)	Most people are basically honest.
13	(Yamagishi 2)	Most people are trustworthy.
14	(Yamagishi 3)	Most people are basically good and kind.

## 4 Study 3

Study 3 was designed to confirm the items and factors identified in Study 2 and provide some evidence that factors identified had construct validity.

### 4.1 Method

**Participants.** One hundred and sixty-six participants were recruited from a large Midwestern University’s undergraduate subjects pool. All participants were recruited through the SONA system and the study was administered through Qualtrics survey. Eight participants were excluded for not completing the study. One hundred and seven were female (68 %) and the mean age was 19.46, *SD* = 1.94.

**Procedure.** Study 3 presented participants with the items in modified instrument presented in Table 3 randomly. Because we had reason to think that this set of 14 items would constitute a reasonable measure of factors of generalized trust, we included other variables to help establish elements of construct validity [47,48]. Participants received the multiple-choice version of the Berlin Numeracy Test [49]. There is no theoretical reason to think that numeracy would be related to trust. Collecting data about numeracy could thereby provide evidence of discriminant validity. Participants also received the Ten Item Personality Inventory that is a brief measure of the Big 5 personality traits [50]. Generalized trust has been shown to be related to agreeableness (sometimes characterized as a facet of agreeableness), openness to experience, and conscientiousness [51]. Replicating the association with personality traits could provide evidence for convergent and discriminant validity, respectively. We also gathered one’s political orientation (1 = strongly liberal, 7 = strongly conservative), age, and sex. Generalized trust has been largely unrelated to sex, political orientation, and age.

### 4.2 Results and discussion

**Fig. 1:**
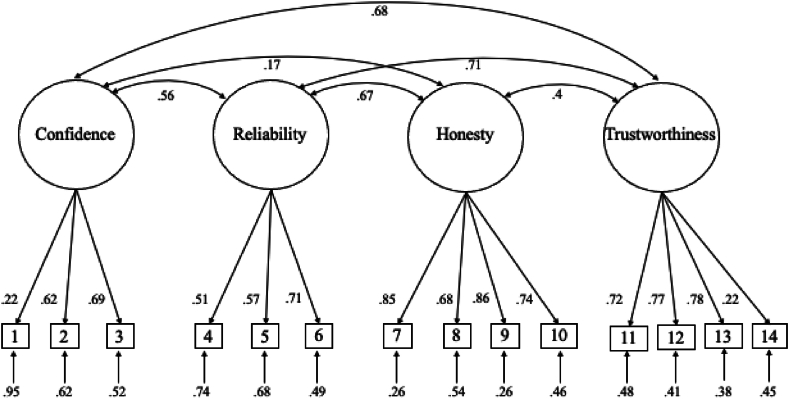
Confirmatory Factor Analysis model with standardized estimates for Study 3. *Note*. The indicator items refer to the items listed in Table 3.

To help verify the factor structure identified in Study 2, a confirmatory factor analysis was conducted specifying the four factors indicated in Table 3 (see Fig. 1 for the structural model). The hypothesized model had acceptable fit to the data fit χ^2^ (71) = 72.67, *p* = .42, *RMSEA* = .01, *RMSEA 90 % CI* 0 - .05, *RMSEA p* = .96, *GFI* = .94, *CFI* = .99, *TLI* = .99.^1^

Correlations among the dependent variables were also calculated (see Table 4). The correlations among trust subscales and personality variables were small to moderate and consistent with results in the literature suggesting convergent validity (e.g., trust is a facet of agreeableness, and we see all 4 general trust factors related to agreeableness). Trust was also not strongly related to sex, age, political orientation, or numeracy suggesting discriminant validity of the trust factors.

**Table 4:** Pearson Correlations among dependent variables for Study 3.

	1	2	3	4	5	6	7	8	9	10	11	12	13
1. Confidence	1												
2. Reliability	.4∗∗	1											
3. Honesty	.14	.5∗∗	1										
4. Trustworthy	.45∗∗	.51∗∗	.34∗∗	1									
5. Extraversion	.31∗∗	.25∗∗	.03	.26∗∗	1								
6. Agreeableness	.29∗∗	.29∗∗	.28∗∗	.24∗∗	−.1	1							
7. Conscientious	.13	.21∗∗	.22∗∗	.18∗	.12	.26∗∗	1						
8. Emotions	.24∗∗	.33∗∗	.04	.21∗∗	.22∗∗	.07	.19∗	1					
9. Openness	.22∗∗	.27∗∗	.16	.25∗∗	.23∗∗	.23∗∗	.21∗∗	.09	1				
10. BNT	.05	.14	.07	.05	.06	−.01	.04	−.01	−.01	1			
11. Age	.07	−.01	.1	.24∗∗	.02	.03	−.07	.07	−.16∗	.04	1		
12. Sex	−.03	−.01	−.06	.07	.12	−.17∗	−.03	.35∗∗	−.15	.02	−.07	1	
13. Politics	−.07	−.13	−.17∗	−.2∗	.03	−.11	.15	.07	−.21∗∗	.01	−.06	.15	1
*Mean*	4.24	3.91	3.04	3.94	3.7	4.46	5.11	4.33	4.97	2.58	19.46		4.04
*SD*	0.75	0.87	0.92	0.81	1.69	1.19	1.17	1.44	1.03	1.07	1.94		1.45

*Note:* ∗*p* < .05, ∗∗*p* < .01.

## 5 Study 4

Study 3 provided evidence for factors identified from Studies 1 and 2 and provided some evidence for scale’s construct validity (e.g., discriminant and convergent validities). Study 4 was designed with two main goals. The first was further validate the factor structure identified in the previous studies. The second was to examine the instrument’s predictive validity in an applied domain of potable recycled water acceptance. Recycled water is water that is treated to a high standard at water reclamation facilities. Then, that water is either directly or indirectly via an environmental buffer transported to drinking water facilities for further treatment and distribution to customers. Both domain general and domain specific trust have been identified as a major factor in recycled water acceptance [[52], [53], [54], [55]]. For that reason, some factors of generalized trust should be related to acceptance of recycled water thereby providing evidence for criterion validity.

### 5.1 Method

**Participants.** Three hundred and thirty-five undergraduates from a large Midwestern University’s subjects pool took part in the study to earn research credits. All participants were recruited through the SONA system and the study was administered through Qualtrics survey. The mean age was 19.52, *SD* = 2.37 and 56 % (*N* = 188) identified as female.

**Procedure.** Participants were first presented with the finalized General Trust scale presented in Table 3. Next, all participants were asked to indicate how comfortable they would feel about drinking recycled water that has been certified safe for drinking and then underwent the following additional treatments [56,57]. These treatments refer to the process of indirect potable reuse.

1. Leaving the water treatment plant, the water travels one mile down a swift river.
2. Leaving the water treatment plant, the water travels one hundred miles down a swift river.
3. Leaving the water treatment plant, the water filters through an underground aquifer for 1 year.
4. Leaving the water treatment plant, the water filters through an underground aquifer for 10 years.
5. Leaving the water treatment plant, the water is deposited in a lake or reservoir for 1 years.
6. Leaving the water treatment plant, the water is deposited in a lake or reservoir for 10 years

Participants responded to each statement using an 11-item scale ranging from 0 = Not comfortable at all to 10 = Completely comfortable. An exploratory factor analysis on these items indicated that there was only 1 factor with an eigenvalue greater than 1. So, we used the mean of responses in analyses.

Next, participants were asked to indicate how comfortable they would feel about drinking recycled water that had been certified safe for drinking. Participants responded to each statement using an 11-item scale ranging from 0 = Not comfortable at all to 10 = Completely comfortable.

Next, Participants were asked to indicate their intention to use recycled water by answering the question “Would you be willing to drink safe certified recycled water?” on an 11-item scale ranging from 0 = Totally unwilling to 10 = Totally willing.

Finally, we collected data on disgust sensitivity (0 = Not disgusted at all, 4 = Strongly disgusted) [58] and we collected data on sex, age, and political identity (1 = Strongly liberal, 7 = Strongly conservative). Previous research has not found that generalized trust is strongly related to disgust sensitivity, sex, or political orientation. However, disgust sensitivity, political orientation, and sex have been associated with recycled water acceptance [53,55,57]. These associations would allow testing whether the factors identified in the trust scale provide incremental predictive validity beyond these known predictors.

### 5.2 Results and discussion

**Fig. 2:**
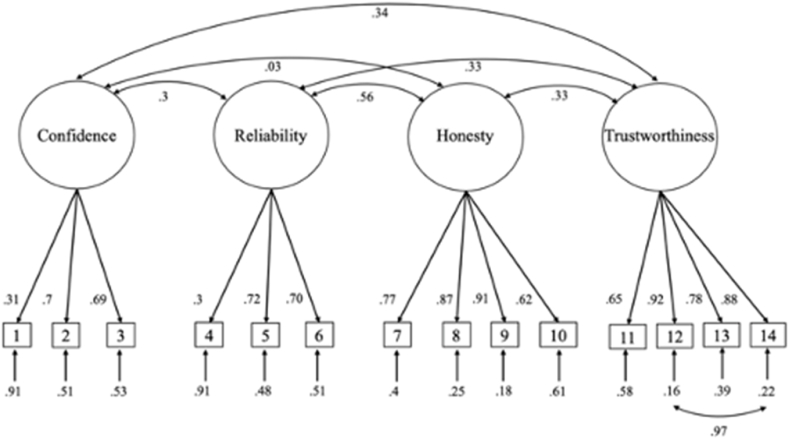
Confirmatory Factor Analysis model with standardized estimates for Study 4. *Note*. The indicator items refer to the items listed in Table 3.

We first attempted to provide evidence for the factor structure identified in the previous studies we call Model 1. A confirmatory factor analysis on the specification in Model 1 revealed an overall moderately good fit to the data χ^2^(71) = 119.83, *p* < .01, *RMSEA* = .05 *90 % CI* [.03 - .06], *RMSEA p* = .68, *GFI* = .95, *CFI* = .97, *TLI* = .96. Here, χ^2^ was significant and the lower bound of the RMSEA did not include 0, indicating some misfit [59]. Some of the model misfit can be attributed to correlated error variance between items 12 and 14 in the final instrument, which is not surprising given the semantic similarity of the items. Allowing those errors to covary in Model 1∗ resulted in overall acceptable model fit: χ^2^(70) = 80.35, *p* = .19, *RMSEA* = .02 *90 % CI* [0 - .04], *RMSEA p* = 1, *GFI* = .97, *CFI* = .97, *TLI* = .96 (see Fig. 2 for the structural model).

We compared the preferred 4 factor model against 3 alternative models (see Table 5). All the alternative models had worse fit [59]. In all the alternative models, we allowed the error variances of items 12 and 14 to covary just as we did with the modified preferred Model 1∗. The first alternative Model 2 included 1 higher order factor including all 4 individual trust factors. While the absolute values of Model 2 might suggest acceptable fit, a likelihood ratio test on the χ^2^ values between Model 1∗ and Model 2 suggested Model 1∗ had a better fit, Δχ^2^ = 23.31, Δdf = 2, *p* < .01. A The second alternative Model 3 included only 1 factor, again with worse fit. The third alternative Model 4 included just two factors. One factor was identified with the trustworthiness items (#s 11, 12, 13, 14) and the other factor was identified by the remaining trust items. This model was meant to estimate the distinction between trusting as a property of the trustor and trustworthiness as a property of the trustee. The third model also had overall worse fit than the preferred 4 factor model.

**Table 5:** Fit indices for the confirmatory factor analyses in Study 4 in order specified.

Model	*χ*^*2*^	*Df*	*χ*^*2*^*p*	*RMSEA*	*RMSEA p*	*GFI*	*CFI*	*TLI*
1	119.83	71	<.01	.05 [.03, .06]	.68	.95	.97	.96
1∗	80.35	70	.19	.02 [0, .04]	1	.97	.97	.96
2	103.86	72	<.01	.04 [.02, .05]	.93	.95	.95	.89
3	841.48	77	<.01	.17 [.16, .18]	<.01	.68	.55	.47
4	338.32	76	<.01	.1 [.09, .11]	<.01	.86	.84	.81

*Note*. Model number = specified model, RMSEA = root mean square error of approximation [90 % confidence interval], GFI = goodness of fit index, CFI = comparative fit index, TLI = Tucker-Lewis Index.

Correlations among the dependent variables were calculated (See Table 6). As was observed in Study 3, the trust factors were not strongly related to age, sex, and or political orientation. Disgust sensitivity was also not strongly related to the trust factors. These results provide additional evidence for discriminate validity of the factors of the general trust scale. Consistent with previous research, responses to the recycled water scale were related to some of the trust factors. Reliability and Trustworthiness were significantly associated with acceptance of indirect potable reuse water and willingness to drink recycled water providing some evidence for predictive validity.

**Table 6:** Pearson correlations among the variables in Study 4.

	1	2	3	4	5	6	7	8	9	10
1. Confidence	1									
2. Reliability	.15∗∗	1								
3. Honesty	−.02	.46∗∗	1							
4. Trustworthy	.24∗∗	.24∗∗	.27∗∗	1						
5. Drink	.07	.17∗∗	.05	.12∗	1					
6. IPR	−.04	.16∗∗	.16∗∗	.13∗	.45∗∗	1				
7. Age	−.08	−.08	−.15∗∗	−.08	−.03	.05	1			
8. Sex	.09	.01	.06	.01	−.14∗∗	−.19∗∗	−.12∗	1		
9. Politics	.07	.04	−.09	−.05	−.17∗∗	−.08	.08	−.19∗∗	1	
10. Disgust	.16∗∗	−.08	−.09	.03	−.2∗∗	−.24∗∗	−.02	.34∗∗	−.02	1
*Mean*	4.59	3.98	2.94	3.7	7.05	4.11	19.52		4	2.24
*SD*	0.74	0.86	0.96	0.85	2.71	3.27	2.37		1.45	0.57

*Note.* Drink = willingness to drink direct potable reuse water. IPR = acceptance of indirect potable reuse water*.* Disgust = disgust sensitivity *∗ p* < .05*, ∗∗p* < .01.

To determine if the Reliability and Trustworthiness factors were unique predictors of acceptance of recycled water, we conducted multiple linear regression with other factors that were correlated with acceptance of recycled water (and have been correlated with acceptance of recycled water in past research [55]). We constructed 4 different multiple regressions (see Table 7). In one set of regressions, willingness to drink recycled water was the outcome variable. In another set of regressions, response to the indirect potable reuse scale was the outcome variable. We then ran multiple regressions using Reliability as a predictor and a separate set of regressions using Trustworthiness as a predictor. In all 4 multiple regressions, we included gender, politics, and disgust sensitivity. After inducing these other variables, Reliability and Trustworthiness remained significant, unique predictors of recycled water acceptance. These results suggest that factors in the general trust scale have criterion validity and can be unique predictors of acceptance of recycled water.

**Table 7:** Multiple regressions predicting drinking recycled water and indirect recycled water acceptance.

Full Model			Predictors					
Reliability and Drink Recycled Water								
*R*^*2*^_*ad*_*j*	*F*	*p*		*B*	*SE*	*β*	*t*	*p*
.11	10.81	<.01	Reliability	0.54	0.16	.17	3.31	<.01
			Politics	−0.4	.1	−.21	4	<.01
			Disgust	−0.03	0.01	−.15	2.63	<.01
			Sex	−0.75	0.31	−.14	2.44	.02
*Reliability and Acceptance of Indirect Potable Reuse Water*								
*R*^*2*^_*ad*_*j*	*F*	*p*		*B*	*SE*	*β*	*t*	*p*
.1	9.82	<.01	Reliability	0.44	0.15	.16	2.97	<.01
			Politics	−0.2	0.09	−.12	2.26	.02
			Disgust	−0.03	.01	−.17	3.1	<.01
			Sex	−0.78	0.27	−.16	2.82	<.01
*Trustworthy and Willingness to Drink Direct Potable Reuse Water*								
*R*^*2*^_*ad*_*j*	*F*	*p*		*B*	*SE*	*β*	*t*	*p*
.1	9.22	<.01	Trustworthy	0.38	0.17	.12	2.27	.02
			Politics	−0.37	0.1	−.2	3.68	<.01
			Disgust	−0.03	.01	−.17	.301	<.01
			Sex	−0.69	0.31	−.13	2.22	.03
*Trustworthy and Acceptance of Indirect Potable Reuse Water*								
*R*^*2*^_*ad*_*j*	*F*	*p*		*B*	*SE*	*β*	*t*	*p*
.09	9.25	<.01	Trustworthy	0.39	0.15	.14	2.6	<.01
			Politics	−0.17	0.09	−.1	1.96	.05
			Disgust	−0.03	0.01	−.19	3.47	<.01
			Sex	−0.72	0.28	−.15	2.63	<.01

## 6 Discussion

Our studies aimed to compare and synthesize commonly used domain general trust measures. Our studies were able to measure 4 interpretable factors of domain general trust: confidence in others, other’s reliability, other’s honesty, and trustworthiness. Results also showed evidence for the instrument’s predictive, convergent, and discriminant validity. Evidence for predictive validity was provided by three out of four factors, i.e., other’s reliability, other’s good intention, and trustworthiness, being significantly positively correlated with participants’ intention to utilize indirect potable reuse. Two out of four factors, i.e., other’s reliability and trustworthiness, were found significantly positively correlated with participants’ willingness to drink safe recycled water. Some evidence for convergent validity was provided by relations to personality traits (e.g., agreeableness). Finally, discriminant validity was provided by being largely unrelated to political orientation, sex, disgust sensitivity, and numeracy.

Our results are consistent with a meta-analysis that looked at different measures of trust as they were related to predicting organizational outcomes like job performance [60]. According to that meta-analysis, there were four trust factors that roughly correspond to the factors we have identified in our scale (our factors indicated in parentheses): Ability (Reliability), benevolence (Confidence), integrity (Honesty), and trust propensity (Trustworthiness). In the meta-regressions, these trust factors moderately predicted job performance, risk taking behaviors, citizenship behaviors, and counterproductive behaviors (*rs* between −.28 and .38). Moreover, the correlations among the factors they identified were roughly similar to the correlations among our factors (correlations among ability, benevolence and integrity were between .62 and .68, and correlations between trustworthiness were between .15 and .29). One benefit of our scale is that rather than meta-analytically reconstructing these factors from data, our 4-factor General Trust scale provides researchers a direct way to measure those factors.

One element that was different from Colquitt et al. [60] study was that they described the 4 factors as “trust antecedents”. They also coded for a “trust” factor that represents a general trusting attitude towards another. They found that this general trust factor meditates the relationships between the trust antecedents and outcome variables. These results may suggest that there is a higher-order factor of “trust” that our four factors contribute to. While the higher-order factor model in Studies 3 and 4 fit the data worse than a simpler 4-factor model, the values of some of the fit indices may suggest that a higher-order factor structure is viable. Future work can help address the utility of having a higher-order model for trust.

These results suggest that there may be some utility using the General Interpersonal Trust Scale. Trust has been identified as being important for a host of decisions ranging from health [61], finance [62], organizations [60], and natural resource management [57] just to name a few domains. At a minimum, our data may provide some guidance about which trust measure to use given the purpose of the study. To offer an illustrative example, Study 4 suggested that some factors of trust are more strongly related to acceptance of recycled water than others. Honesty was more strongly to recycled water acceptance (*r* = .16) than Confidence (*r* = −.04), a significant difference (*Steiger’s z* = 3.54, *p* < .01, *Cohen’s Q* = .2). This difference in correlations could therefore influence the estimates of the strength of the relations between trust and outcome variables. If items measuring only confidence were used, one would infer no reliable relation between trust and recycled water acceptance. If Honesty were used, one would infer a reliable relation. Hence, our data can help to start to identify and document which elements of trust are related to different outcomes.

We think there are several potential paths forward. First, we want to emphasize that the process of instrument validation is a complicated and long process. No set of a few studies can fully explore and validate an instrument [48]. Rather, we take our studies as a first step. To illustrate, we only used U.S participants and only had a handful of variables to help establish construct validity. Future work should establish how well the instrument performs on different groups of people (e.g., invariance testing) and different variables (e.g., risk perceptions; affective reactions). Additionally, we only tested this instrument with one outcome variable (i.e., intention to use recycled water). We acknowledge that using one criterion outcome variable (i.e., recycled water use) is a limitation in our study. Future research should test this trust measure in other contexts and with different outcome variables (e.g., job performance).

It is also worth noting that we focused on *general* rather than *particularized* trust. Given the current data, we have no evidence how, to what extent, and when our instrument is related to particularized trust. For example, we anticipate that in many situations, the domain general trust factors will be mediated by more domain specific trust factors. There is some evidence that this is true. Take for instance acceptance of recycled water. Some research suggests that while generalized trust factors can predict acceptance of recycled water, particularized trust (e.g., trust in water provider) was more strongly related to intentions to drink recycled water [55]. However, by the transitivity, domain general trust should predict domain specific trust which should in turn predict intentions toward recycled water (i.e., highly general trusting individuals might also have high trust in water reuse entities and therefore have higher intention to use potable recycled water). We would expect this general tendency for particularized trust to fully mediate relations found with domain general trust in many contexts. Future research can help establish these relations.

With these potential shortcomings in mind, we hope to have provided a useful next step in understanding and measuring trust. At a minimum, having a comparison of all major, currently existing generalized trust scales may help researchers decide which instruments to use and when. Moreover, our General Interpersonal Trust Scale that measures all four major components of trust may be of use to researchers who wish to estimate all four major factors in a relatively short instrument.

## CRediT authorship contribution statement

**Uyen Hoang:** Writing – review & editing, Writing – original draft, Software, Resources, Methodology, Investigation, Formal analysis, Data curation. **Braden Tanner:** Methodology. **Dana Mahmoud-Elhaj:** Resources. **Jenna Holt:** Resources. **Muhammad Asif:** Resources. **Adam Feltz:** Writing – review & editing, Writing – original draft, Visualization, Validation, Supervision, Software, Resources, Project administration, Methodology, Investigation, Formal analysis, Conceptualization.

## Data availability statement

The data that support the findings of these studies are openly available in Open Science Framework at OSF.IO/PWXTF" title = "https://doi.org/10.17605/OSF.IO/PWXTF">https://doi.org/10.17605/OSF.IO/PWXTF.

## Declaration of competing interest

The authors report there are no competing interests to declare.

## Footnotes

1 Results from a second-order confirmatory factor analysis indicated that the four factors might load onto a higher order general trust factor, χ^2^ (73) = 91.04, *p* = .08, *RMSEA* = .04, *RMSEA 90 % CI* 0 - .06, *RMSEA p* = .74, *GFI* = .99, *CFI* = .98, *TLI* = .97. However, the higher-order trust CFA had a worse fit to the data than the CFA without the higher order trust factor: Δχ^2^ = 18.38, Δdf = 2, *p* < .01.
