# Supplementary material for: Towards a unified measure of general interpersonal trust
Source: Heliyon. 2024 Nov 22;10(23):e40624. doi: 10.1016/j.heliyon.2024.e40624 (PMC11626050; doi:10.1016/j.heliyon.2024.e40624)

**IRT FOR STUDY 1**

**FACTOR 1—OTHER’S RELIABILITY:**

🡺 Original items (*highlighted items are the items retained for further refinement in Study 2*):

| Item | Content |
| --- | --- |
| **Ash R2** | **Other people cannot be relied upon.** |
| **Ash R6** | **Other people who act in friendly way towards me are disloyal behind my back.** |
| **Evans R17** | **I feel shortchanged in life.** |
| **Ash R3** | **I have little faith in other people’s promises.** |
| **Ash R9** | **Other people let you down.** |
| **Couch R20** | **I would admit to being more than a little paranoid about people I meet.** |
| **Rotter 14** | **Most elected officials are really sincere in their campaign promises.** |
| Couch 23 | I am rarely ever suspicious of people with whom I have a relationship. |
| Ash R7 | Other people lie to get ahead. |
| Couch 37 | I feel I can depend on most people I know. |

🡺 IRT Results:

F1 h2

**AshR2 0.826 0.6831**

**AshR6 0.763 0.5826**

**EvansR17 0.510 0.2602**

**AshR3 0.752 0.5651**

**AshR9 0.691 0.4770**

**CouchR20 0.653 0.4258**

**Rotter 14 -0.135 0.0183**

Couch 23 0.593 0.3522

AshR7 0.554 0.3072

Couch 37 0.509 0.2589


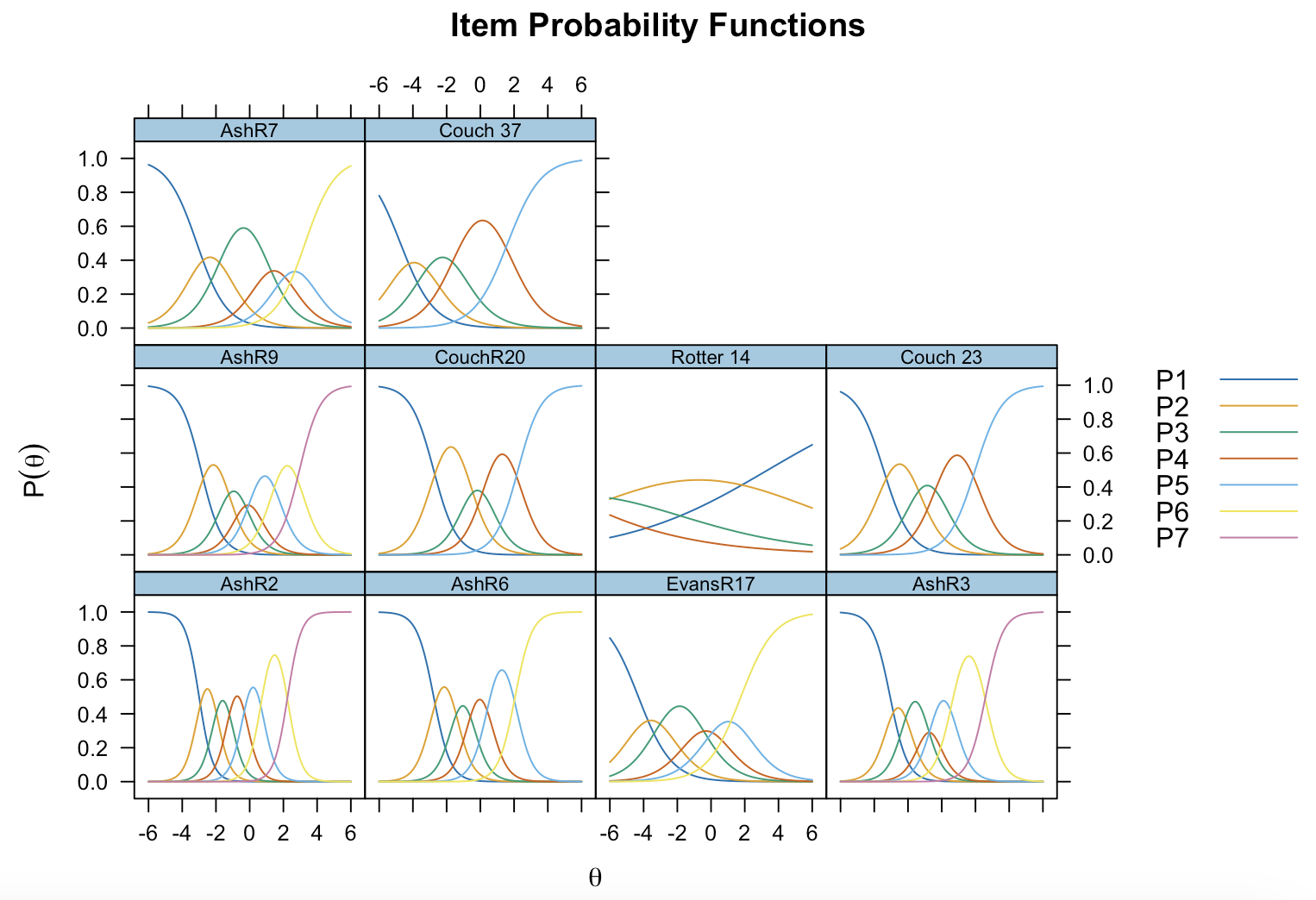


**FACTOR 2—OTHER’S TRUSTWORTHINESS:**

🡺 Original items (*highlighted items are the items retained for further refinement in Study 2*):

| Item | Content |
| --- | --- |
| **Yamagishi 3** | **Most people are basically good and kind.** |
| **Yamagishi 2** | **Most people are trustworthy.** |
| **Yamagishi 1** | **Most people are basically honest.** |
| **Rotter 20** | **Most idealists are sincere and practice what they preach.** |
| **Evans 13** | **I believe that people are basically moral.** |
| **Yamagishi 6** | **Most people will respond in kind when they are trusted by others.** |
| Yamagishi 5 | I am trustful. |

🡺 IRT results:

F1 h2

**Yamagishi 3 0.828 0.686**

**Yamagishi 2 0.926 0.858**

**Yamagishi 1 0.822 0.676**

**Rotter 20 0.472 0.223**

**Evans 13 0.555 0.308**

**Yamagishi 6 0.633 0.400**

Yamagishi 5 0.586 0.344


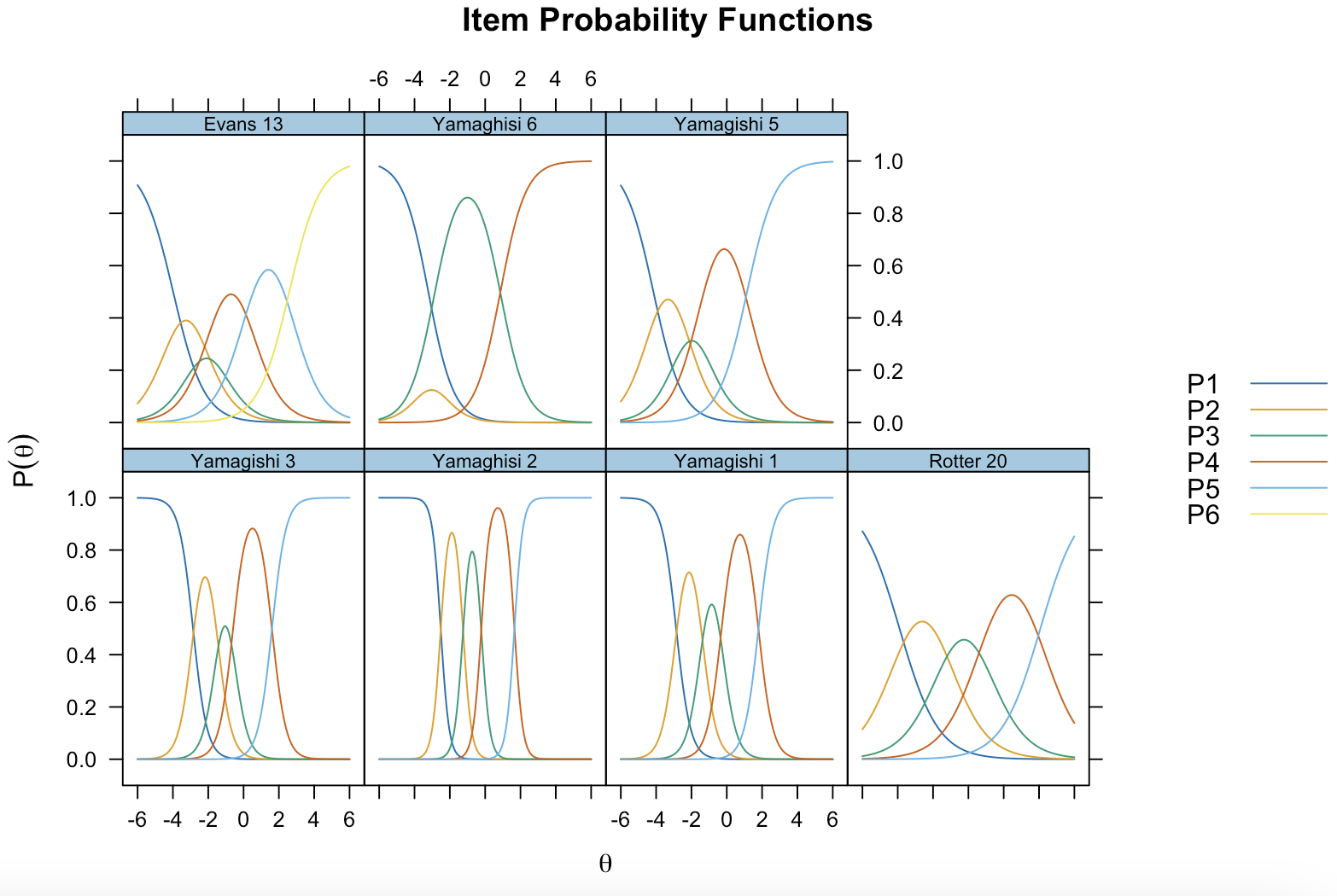


**FACTOR 3—OTHER’S GOOD INTENTION:**

🡺 Original items (*highlighted items are the items retained for further refinement in Study 2*):

| Item | Content |
| --- | --- |
| **Rotter R10** | **It is safe to believe that in spite of what people say most people are primarily interested in their own welfare.** |
| **Ashleigh R8** | **Other people are only concerned with their own well-being.** |
| **Ashleigh R4** | **Other people are primarily interested in their own welfare despite what they say.** |
| **Ashleigh R1** | **Other people are out to get as much as they can for themselves.** |
| **Rotter R19** | **In these competitive times one has to be alert or someone is likely to take advantage of you.** |
| **Rotter 22** | **Most students in school would not cheat even if they were sure of getting away with it.** |
| **WVS 2** | **Most of the time, people try to be helpful.**  **Most of the time, people are mostly looking out for themselves.** |
| Evans R19 | I believe that most people would lie to get ahead. |
| Ash R5 | In these competitive times, I have to be alert; otherwise, others will take advantage of me. |

🡺 IRT Results:

F1 h2

**RR10 0.735 0.541**

**AshR8 0.695 0.483**

**AshR4 0.764 0.583**

**AshR1 0.725 0.525**

**rR19 0.596 0.355**

**Rotter 22 0.422 0.178**

**WVS 2 0.638 0.407**

EvansR21 0.461 0.213

EvansR19 0.516 0.267

AshR5 0.624 0.389


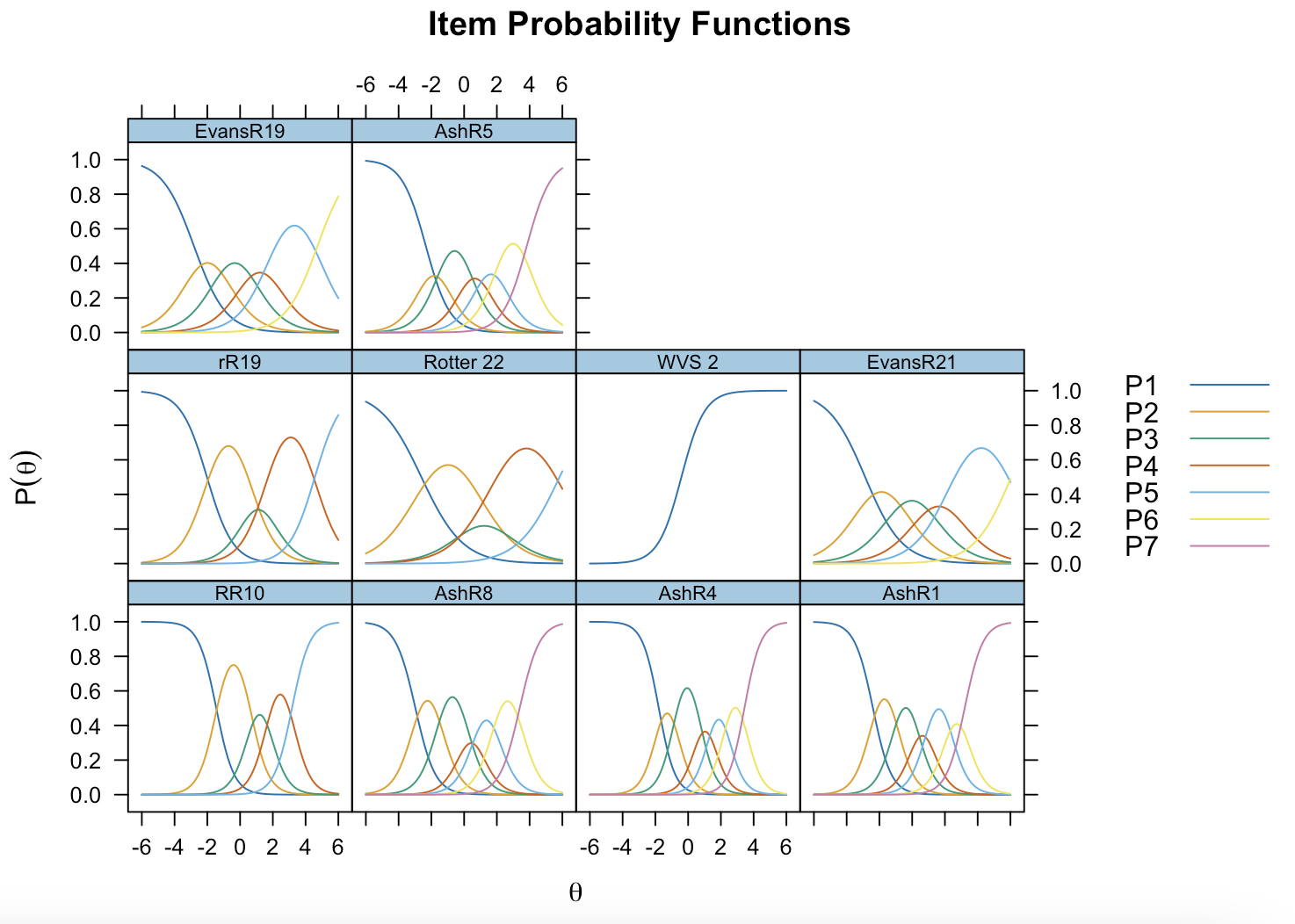


**FACTOR 4—CONFIDENCE IN OTHERS:**

🡺 Original items (*highlighted items are the items retained for further refinement in Study 2*):

| Item | Content |
| --- | --- |
| **Rotter 25** | **Most people answer public opinion polls honestly.** |
| **Evans 4** | **I can get along with most people.** |
| **Evans 8** | **I believe that laws should be strictly enforced.** |
| **Evans 9** | **I value cooperation over competition.** |
| **Couch 2** | **I tend to be accepting of others.** |
| **Couch 5** | **I do not worry that my partner will leave me.** |
| **Couch 30** | **I have a lot of faith in people I know.** |
| **Couch 35** | **When it comes to people I know, I am believing and accepting.** |
| Couch 37 | I feel like I can depend on most people I know. |
| Yamagishi 5 | I am trustful |
| Ashleigh 12 | Other people answer public opinion polls honestly. |

🡺 IRT Results:

F1 h2

**Couch 2 0.677 0.4586**

**Couch 30 0.620 0.3845**

**Couch 35 0.790 0.6243**

**Rotter 25 0.548 0.3002**

**Couch 5 0.310 0.0963**

**Evans 8 0.612 0.3740**

**Evans 4 0.731 0.5343**

**Evans 9 0.607 0.3681**

Couch 37 0.674 0.4542

Yamagishi 5 0.551 0.3038

Ashleigh 12 0.458 0.2099


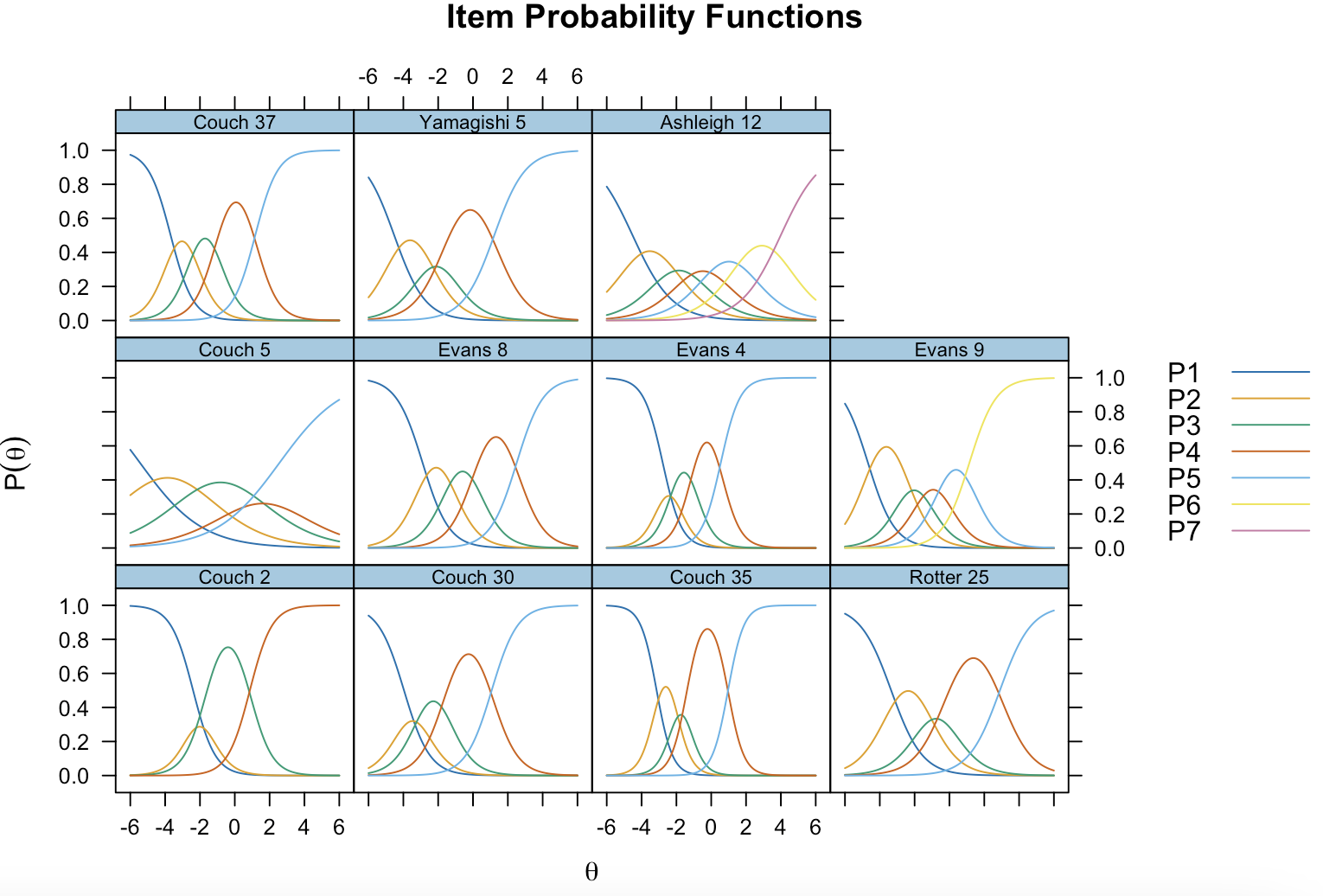

Supplement: Multimedia component 1 [file mmc1.docx]
