# Supplementary material for: Towards a unified measure of general interpersonal trust
Source: Heliyon. 2024 Nov 22;10(23):e40624. doi: 10.1016/j.heliyon.2024.e40624 (PMC11626050; doi:10.1016/j.heliyon.2024.e40624)

**IRT FOR STUDY 2**

**FACTOR 1—OTHER’S RELIABILITY:**

🡺 Original items (*highlighted items are the retained items for the Other’s Reliability Factor in the finalized trust scale*):

| Item | | Origin | Content |
| --- | --- | --- | --- |
| **1** | **Trust 12** | **Ashleigh R2** | **Other people cannot be relied upon.** |
| 2 | Trust 14 | Ashleigh R6 | Other people who act in friendly way towards me are disloyal behind my back |
| **3** | **Trust 11** | **Evans R17** | **I feel shortchanged in life.** |
| 4 | Trust 13 | Ashleigh R3 | I have little faith in other people’s promises. |
| **5** | **Trust 15** | **Ashleigh R9** | **Other people let you down.** |
| 6 | Trust 17 | Couch R20 | I would admit to being more than a little paranoid about people I meet. |
| 7 | Trust 10 | Rotter 14 | Most elected officials are really sincere in their campaign promises. |

🡺 IRT Results:

F1 h2

**Rtrust12 0.9196 0.84559**

Rtrust14 0.7242 0.52444

**Rtrust11 0.6211 0.38582**

Rtrust13 0.8461 0.71590

**Rtrust15 0.7957 0.63312**

Rtrust17 0.7238 0.52390

Trust10 0.0363 0.00131


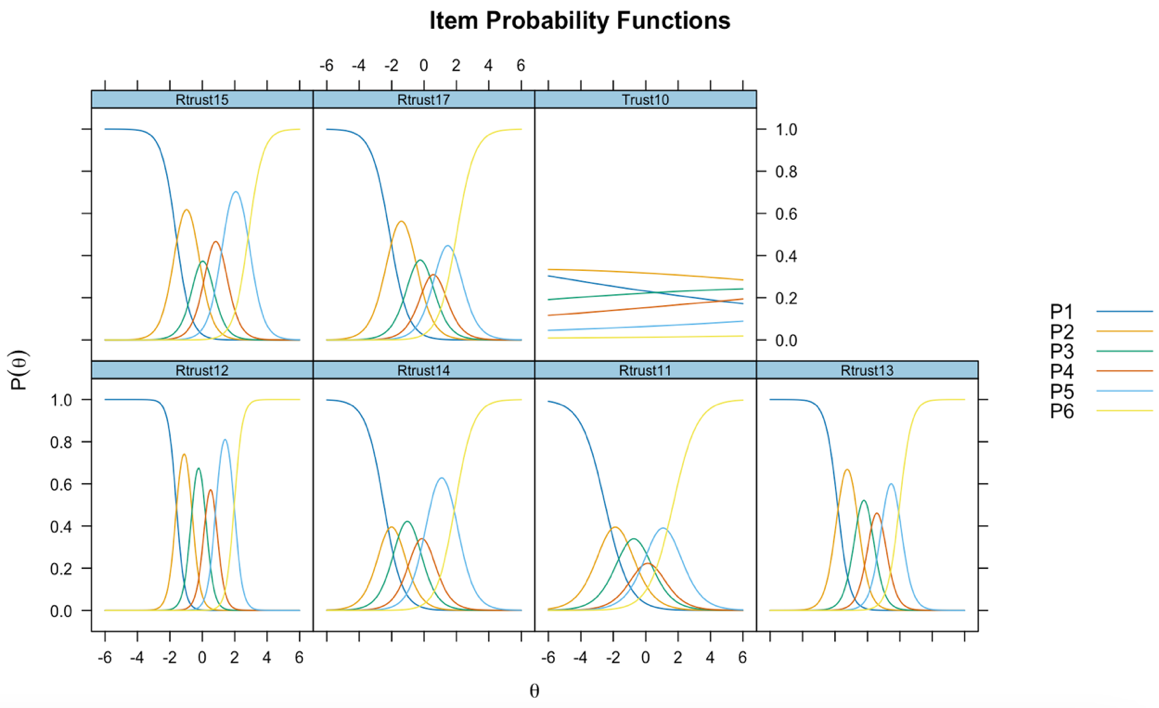


**FACTOR 2—OTHER’S TRUSTWORTHINESS:**

🡺 Original items (*highlighted items are the retained items for the Other’s Trustworthiness Factor in the finalized trust scale*)

| Item | | Origin | Content |
| --- | --- | --- | --- |
| **8** | **Trust 43** | **Yamagishi 3** | **Most people are basically good and kind.** |
| **9** | **Trust 42** | **Yamagishi 2** | **Most people are trustworthy.** |
| **10** | **Trust 41** | **Yamagishi 1** | **Most people are basically honest.** |
| 11 | Trust 39 | Rotter 20 | Most idealists are sincere and practice what they preach. |
| **12** | **Trust 40** | **Evans 13** | **I believe that people are basically moral** |
| 13 | Trust 44 | Yamagishi 6 | Most people will respond in kind when they are trusted by others. |

🡺 IRT results:

F1 h2

**Trust43 0.936 0.876**

**Trust42 0.895 0.800**

**Trust41 0.922 0.850**

Trust39 0.614 0.377

**Trust40 0.858 0.737**

Trust44 0.634 0.402


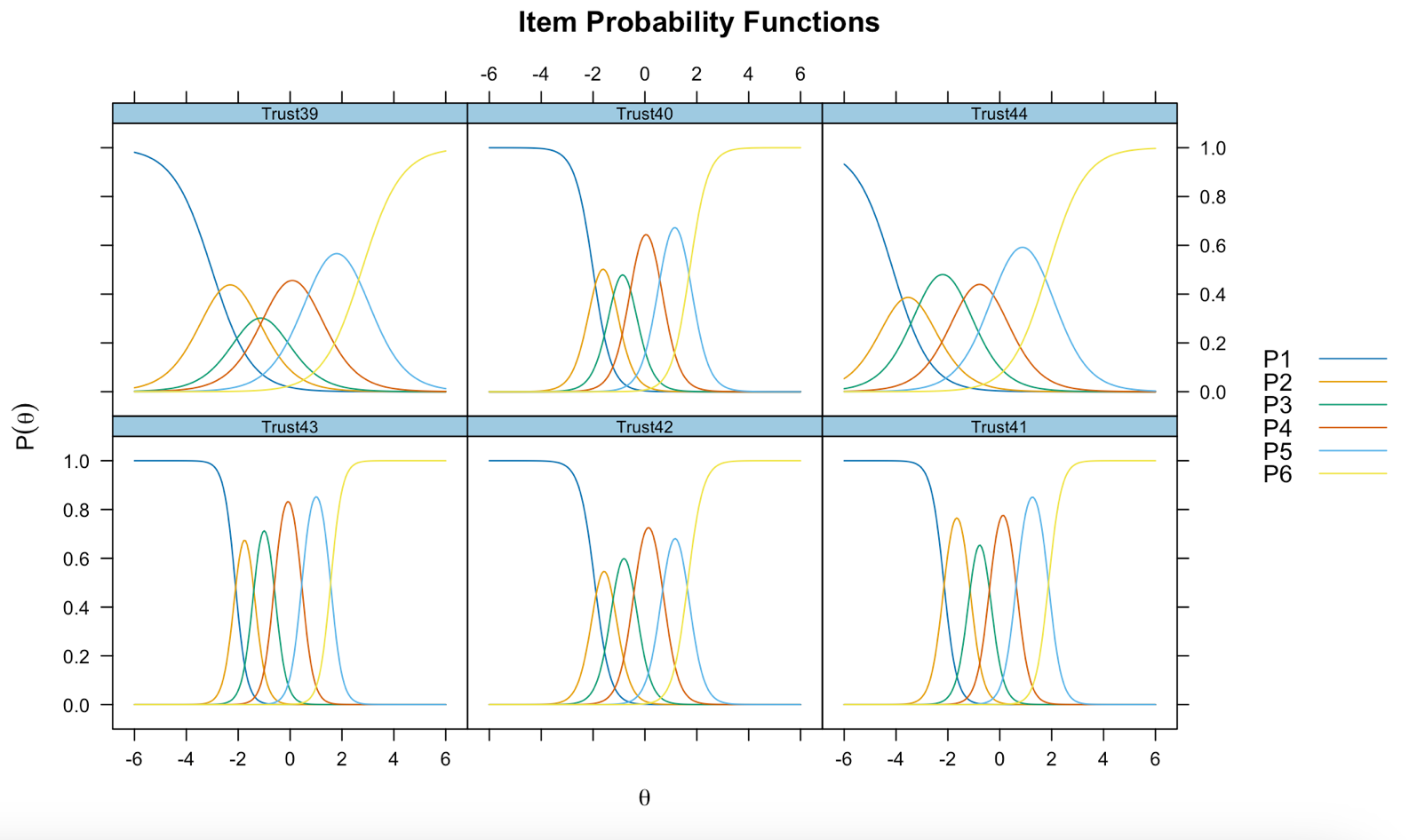


**FACTOR 3—OTHER’S GOOD INTENTION:**

🡺 Original items (*highlighted items are the retained items for the Other’s Good Intention Factor in the finalized trust scale*):

| Item | | Origin | Content |
| --- | --- | --- | --- |
| **14** | **Trust 28** | **Rotter R10** | **It is safe to believe that in spite of what people say most people are primarily interested in their own welfare.** |
| **15** | **Trust 38** | **Ashleigh R8** | **Other people are only concerned with their own well-being.** |
| 16 | **Trust 36** | **Ashleigh R4** | **Other people are primarily interested in their own welfare despite what they say.** |
| 17 | Trust 35 | Ashleigh R1 | Other people are out to get as much as they can for themselves. |
| 18 | Trust 29 | Rotter R1 | In these competitive times one has to be alert or someone is likely to take advantage of you. |
| 19 | Trust 30 | Rotter 22 | Most students in school would not cheat even if they were sure of getting away with it. |
| **20** | Trust 33  **Trust 34** | WVS2_1  **WVS2_2** | Most of the time, people try to be helpful.  **Most of the time, people are mostly looking out for themselves.** |

🡺 IRT Results:

F1 h2

**Rtrust28 0.911 0.8296**

**Rtrust38 0.820 0.6723**

**Rtrust36 0.911 0.8302**

Rtrust35 0.827 0.6833

Rtrust29 0.773 0.5979

Trust30 0.203 0.0412

Trust33 0.247 0.0610

**Rtrust34 0.895 0.8008**


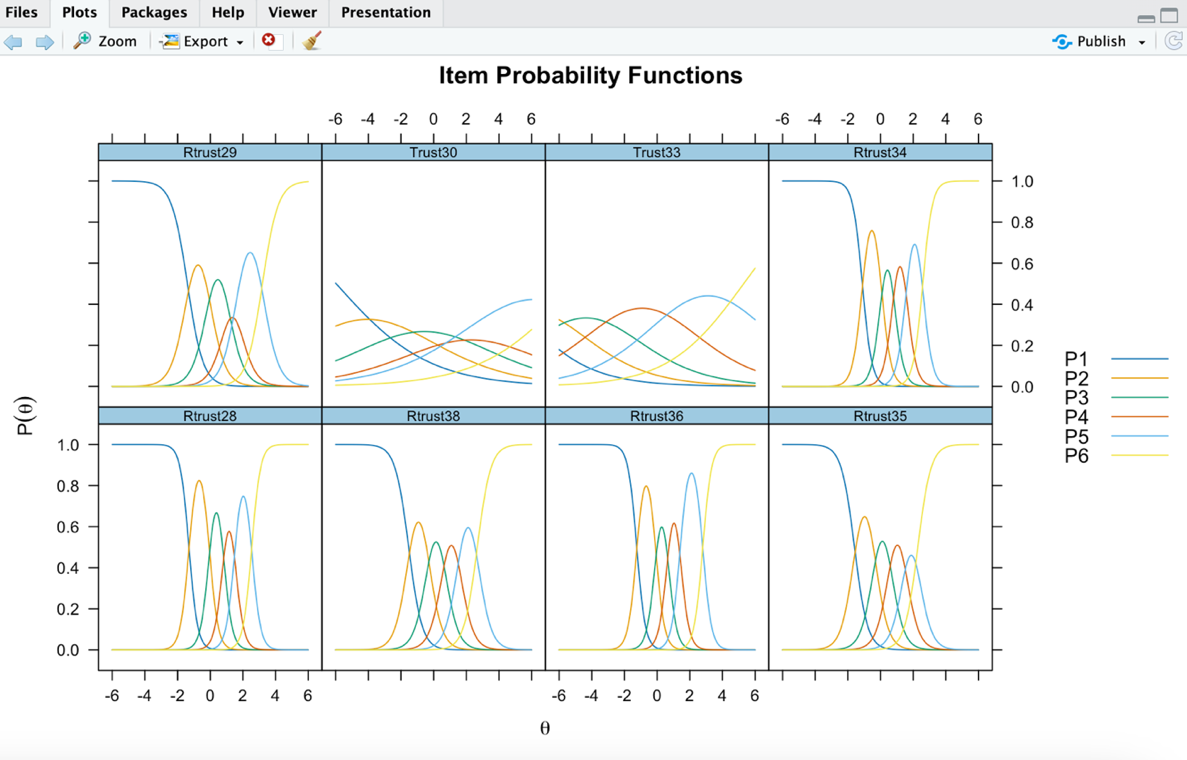


**FACTOR 4—CONFIDENCE IN OTHERS:**

🡺 Original items (*highlighted items are the retained items for the Confidence in Others Factor in the finalized trust scale*):

| Item | | Origin | Content |
| --- | --- | --- | --- |
| **21** | **Trust 6** | **Couch 2** | **I tend to be accepting of others.** |
| 22 | Trust 8 | Couch 30 | I have a lot of faith in people I know. |
| 23 | Trust 9 | Couch 35 | When it comes to people I know, I am believing and accepting. |
| **24** | **Trust 1** | **Rotter 25** | **Most people answer public opinion polls honestly.** |
| 25 | Trust 7 | Couch 5 | I do not worry that my partner will leave me. |
| 26 | Trust 3 | Evans 8 | I believe that laws should be strictly enforced. |
| **27** | **Trust 2** | **Evans 4** | **I can get along with most people.** |
| 28 | Trust 4 | Evans 9 | I value cooperation over competition. |

🡺 IRT Results:

F1 h2

**Trust1 0.584 0.3413**

**Trust2 0.650 0.4221**

Trust 3 0.288 0.0827

Trust4 0.521 0.2719

**Trust6 0.629 0.3957**

Trust7 0.288 0.0831

Trust8 0.862 0.7425

Trust9 0.871 0.7582


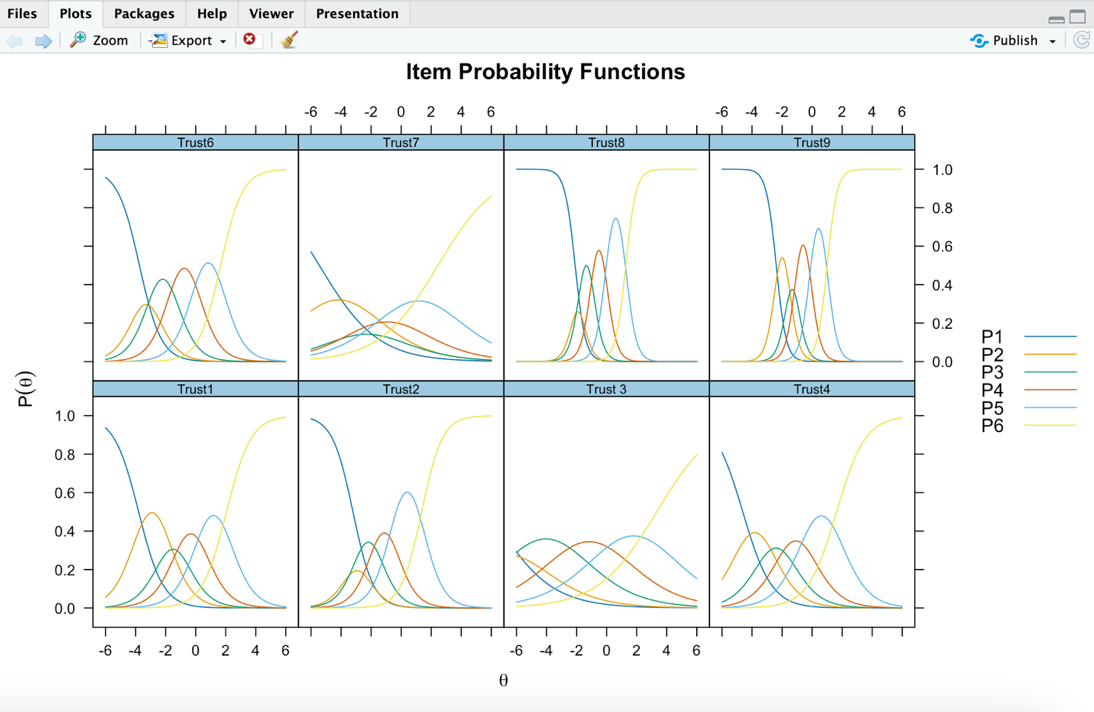

Supplement: Multimedia component 2 [file mmc2.docx]
